# Coupled Mechanisms of Pore–Throat Structure Regulation and Flow Behavior in Deep-Water Tight Reservoirs Using Nanocomposite Gels

**DOI:** 10.3390/gels12020113

**Published:** 2026-01-28

**Authors:** Yuan Li, Fan Sang, Guoliang Ma, Hujun Gong

**Affiliations:** 1 Third Gas Production Plant of Yanchang Gas Field, Shaanxi Yanchang Petroleum (Group) Co., Ltd., Yan’an 717500, China; 15863241782@163.com; 2 Huanqing Oil Production Plant, Yumen Oilfield, Qingyang 745700, China; 18973264792@163.com; 3 Huanqing Oil Production Plant, Yumen Oilfield Branch of China National Petroleum Corporation (PetroChina), Jiuquan 735000, China; 15642321564@163.com; 4 Department of Geology, Northwest University, Xi’an 710069, China

**Keywords:** nanocomposite gel, pore–throat reconstruction, interfacial chemical properties, micro/nano-scale pore characterization, flow-path regulation, tight reservoirs, conformance control

## Abstract

Understanding how nanocomposite gels regulate pore–throat structures and flow behavior is essential for improving profile control and flow diversion in deep-water tight reservoirs. In this study, a dual-structure-regulated nanocomposite gel (DSRC-NCG) was designed, and its structure–flow coupling behavior during gel injection, curing, and degradation was systematically investigated using multiscale flow configurations, including microfluidic models, artificial cores, and sandpack systems. Microstructural evolution and pore–throat connectivity were characterized using μCT imaging, mercury intrusion porosimetry, nitrogen adsorption, and image-based flow simulations, while macroscopic flow responses were evaluated through permeability variation, dominant-channel evolution, injectivity behavior, and quantitative indices including the structure regulation index (SRI) and pore–flow matching index (HCI). The results show that increasing SiO_2_ content induces a progressive optimization of pore–flow matching by refining critical throats and suppressing preferential flow channels, whereas excessive nanoparticle loading leads to aggregation and attenuation of these effects. This study proposes a multiscale structure–flow coupling framework that quantitatively connects pore–throat regulation with macroscopic flow responses during nanocomposite gel injection and degradation. These findings offer mechanistic insights and practical guidance for the design of nanocomposite gels with improved flow-regulation efficiency and reversibility in deep-water tight reservoir applications.

## 1. Introduction

Deep-water tight reservoirs are characterized by ultralow porosity and permeability, strong heterogeneity, and complex multiscale pore–throat architectures, all of which have long constrained effective stimulation and stable production. Numerous studies have shown that the pore systems of such reservoirs are dominated by micropores and mesopores, while their pore–throat networks exhibit pronounced fractal and hierarchical features. Connectivity is highly dependent on a limited number of dominant channels, resulting in extremely focused flow pathways and pronounced sensitivity to completion fluids and plugging agents [1,2,3]. Systematic characterization using MICP, N_2_ adsorption, and μCT has revealed the pore structure, throat-size distribution, and connectivity of deep-water and continental tight sandstones, highlighting a multilevel coupling pattern of “micropore storage–mesopore transition–fine-throat flow control” and clarifying pore–throat evolutionary behaviors under varying differential pressure, salinity, and stress conditions [4,5,6,7]. Beyond purely structural description, recent reservoir-engineering studies have emphasized that micro-scale pore–throat attributes directly govern seepage regimes, permeability evolution, and flow nonlinearity in tight reservoirs. In particular, quantitative analyses have demonstrated that variations in throat size distribution, coordination number, and dominant-channel continuity can induce orders-of-magnitude changes in effective permeability and micro-scale seepage characteristics, even when porosity remains nearly constant.

These findings underscore that pore–throat structure is not only a microscopic descriptor but also a fundamental control parameter linking material-scale modification to macroscopic permeability response and reservoir productivity, providing an essential bridge between pore-scale characterization and field-scale flow behavior. However, these studies primarily focus on natural pore–throat features, and quantitative insights into the coupling among chemical regulation, structural response, and flow-path reconstruction remain limited.

Polymer/weak-gel profile-control and water-plugging systems have been extensively applied in high-water-cut oil and gas reservoirs to mitigate water channeling, fingering, and post-stimulation instability in deep-water tight formations [8,9,10,11]. Previous research has investigated polyacrylamide-based gels [12,13], organically crosslinked Cr/Al gels [14,15], and three-dimensional network elastic gels [16] in terms of formulation optimization, thermal stability, salt sensitivity, and mechanical performance, confirming their effectiveness in enhancing sweep efficiency and suppressing high-permeability channels. Nonetheless, traditional gel systems often suffer from crosslink-ageing, insufficient volumetric stability, and poor injectivity in fine pore–throat networks under high-temperature and high-salinity conditions, leading to “strong plugging but easy damage”, near-well overblocking, and secondary formation impairment. These limitations make them poorly suited for deep-water tight reservoirs, where strong stress fields, high salinity, and ultrafine pore–throat structures coexist [17,18].

In recent years, nanoparticle-reinforced composite gels have emerged as a promising direction for reservoir regulation and completion fluids due to their designable microstructures, high specific surface area, and tunable interfacial properties. Studies have demonstrated that incorporating SiO_2_, TiO_2_, clay nanosheets, or organic nanomicelles into polymer networks can significantly improve gel thermal stability, shear resistance, and mechanical strength while enhancing migration and retention within fine pore–throat systems [19,20,21,22]. Although SEM, AFM, NMR, DMA, and rheological analyses have clarified how nanofiller content, surface modification, and crosslink density affect gel network structure and material-scale mechanics, most research remains focused on the relationship between bulk structure and macroscopic properties.

Recent advances in reservoir-scale modeling further suggest that microstructural regulation of pore–throat systems can propagate upward to influence macroscopic permeability and flow efficiency through multiscale coupling mechanisms [23,24]. Hybrid-dimensional and dual-porosity–dual-permeability models explicitly incorporate pore–fracture or pore–cavity flux interactions, demonstrating that micro-scale structural modification can significantly alter effective permeability tensors and flow partitioning in heterogeneous reservoirs.

Systematic understanding of how such gels achieve selective occupation, controllable plugging, and reversible unblocking within real tight-rock pore networks is still lacking [25,26]. Recent studies have further extended nanoparticle-reinforced composite gels toward enhanced oil recovery and reservoir conformance applications. It has been reported that polymer–nanoparticle composite systems can improve sweep efficiency and flow conformance by strengthening gel networks, modifying interfacial wettability, and selectively restricting high-permeability channels under reservoir conditions [27,28,29]. These macroscopic performance improvements are increasingly interpreted as emergent outcomes of pore–throat-scale structural regulation, dominant-channel redistribution, and permeability rebalancing across heterogeneous flow units. However, existing studies predominantly emphasize macroscopic recovery performance or qualitative plugging behavior, while quantitative understanding of how nanofiller-induced structural regulation translates into pore–throat reconstruction and multiscale flow responses remains insufficient, particularly for deep-water tight reservoirs.

On the pore-scale characterization front, X-ray μCT and microfluidic visualization techniques have provided critical tools for examining gel–porous media interactions. Some studies using μCT have mapped the three-dimensional distribution of polymer gels within sandstone cores and revealed mechanisms of relative permeability alteration, suggesting that gels preferentially invade high-permeability channels and form “bridging–constriction” structures at pore–throat intersections, thereby explaining asymmetric oil–water permeability responses [30,31,32,33]. Other researchers have used glass microfluidic chips to visualize displacement processes of polymer gels and nanogel systems, observing transitions from “coarse continuous channels” to “multibranch fine networks” during injection and proposing the concept of “dominant-channel redistribution” [34,35]. However, these studies typically focus on moderate permeability rocks or ambient conditions and often rely on a single characterization technique, making it difficult to quantify multiscale relationships among pore–throat parameters, interfacial chemistry, mechanical stability, and flow responses within a unified framework.

A synthesis of existing literature reveals three major gaps:

(1) Nanocomposite gel systems tailored for the extreme conditions of deep-water tight reservoirs (high temperature, high salinity, strong confinement) remain scarce, and few studies have systematically examined the quantitative links between material structural parameters and pore–throat regulatory behavior.

(2) Cross-scale coupling research on gel structure/composition–pore network reconstruction–flow behavior evolution is insufficient; most studies address plugging or profile control at a single scale or under a single scenario, lacking continuous evidence chains that span from material microstructure to three-dimensional pore networks and macroscopic permeability responses.

(3) Evaluation systems for reversible or degradable gels—particularly regarding their “plugging–unplugging” processes and reversible pore–throat regulation—have not been established, leaving the mechanisms underlying low-damage and highly controllable completion fluids inadequately understood.

To address these gaps, this study proposes a multiscale experimental and analytical framework—from material structure to pore–throat regulation and seepage behavior—based on the nanocomposite gel DSRC-NCG. The work proceeds across three interconnected levels. First, at the material scale, SEM/AFM, FTIR/XPS, rheological and mechanical tests, and N_2_ adsorption are employed to quantitatively characterize the effects of nano-SiO_2_ content on gel multiscale pore structure, interfacial chemistry, and mechanical stability. A structural regulation index (SRI) is proposed to collectively evaluate the synergistic effects of throat refinement, distribution convergence, and specific surface area enhancement. Second, at the rock and model scales, microfluidic devices, multigrade-permeability artificial cores, and dry-gel-coated sandstone packings are used to conduct combined flow experiments. Indicators such as the pore–flow matching index (HCI) and residual resistance factor (RRF) are introduced to systematically assess the capacity of nanocomposite gels to regulate dominant channels and cross-permeability pathways under different heterogeneity conditions. Third, at the in situ μCT scale, time-sequence scanning and lattice Boltzmann inversion are used to obtain network parameters including connected porosity *φ*_c_, dominant-channel volume fraction *V*_dom_, coordination number *Z*, tortuosity *τ*, and Euler characteristic *χ*, establishing quantitative relationships between pore–throat evolution and seepage capability (*k*_img_, *k*_exp_) during gel injection, curing, and degradation.

Through these multiscale characterizations and flow experiments, this study aims to identify the optimal compositional window of DSRC-NCG under deep-water tight reservoir conditions and elucidate the mechanisms enabling dominant-channel reconstruction, preferential occupation of fine pores, and reversible plugging. The results provide an integrated materials–structure–flow framework for designing completion fluids and profile-control systems for deep-water tight reservoirs, offering theoretical foundations and parameter references for low-damage and high-efficiency reservoir stimulation. Therefore, this study aims to design a dual-structure-regulated nanocomposite gel for deep-water tight reservoirs and to quantitatively reveal the coupling between gel structural regulation, pore–throat reconstruction, and multiscale flow behavior during injection and degradation.

## 2. Results and Discussion

### 2.1. Structural and Performance Characterization of the Gel

#### 2.1.1. Multiscale Network Structure and Morphological Evolution

**Figure 1:**
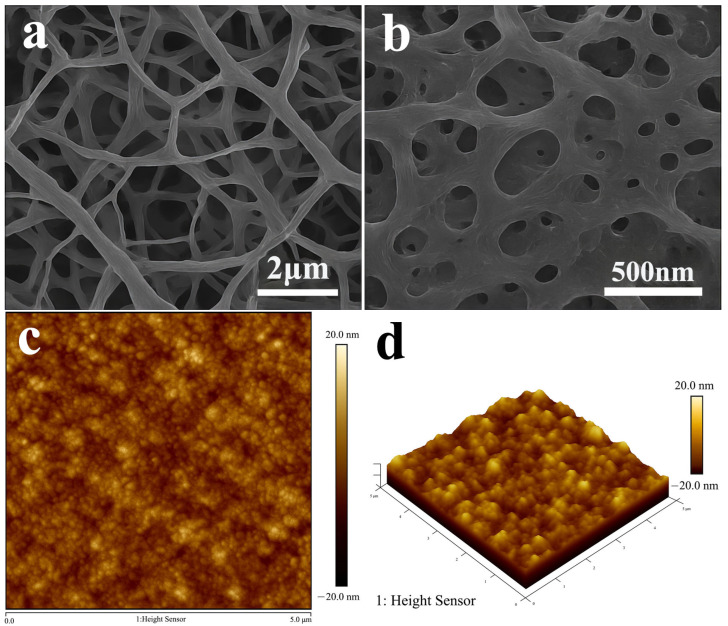
SEM and AFM characterization of the microstructure of DSRC-NCG. (**a**) SEM image (×10,000) showing the porous network and micron-scale pore distribution (scale bar: 2 µm); (**b**) high-magnification SEM image (×30,000) revealing pore-wall details with embedded SiO_2_ nanoparticles (scale bar: 500 nm); (**c**) AFM 2D height map (scan area: 5 × 5 µm^2^, height scale: −20 to 20 nm) showing nanoscale roughness distribution; (**d**) AFM 3D height map (scan area: 5 × 5 µm^2^, height scale: −20 to 20 nm) illustrating nanoscale protrusions and groove morphology.

**Figure 2:**
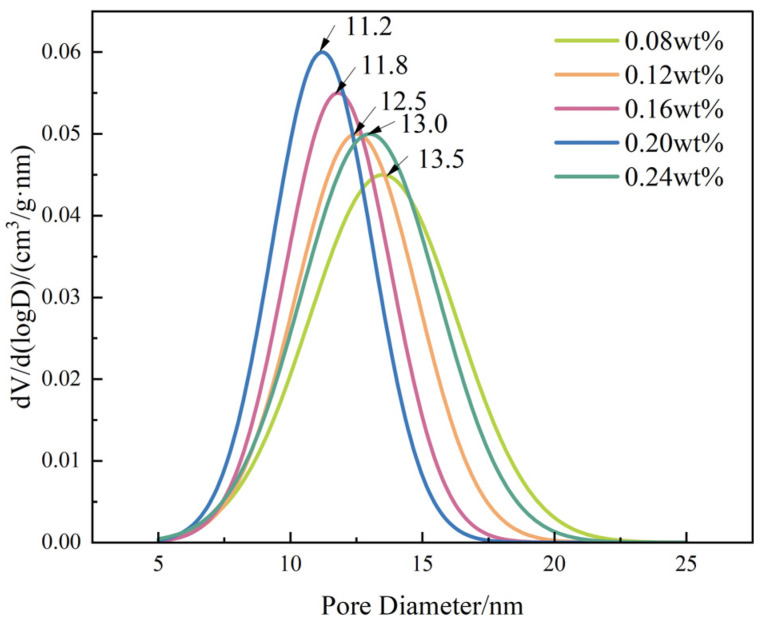
Pore-size distribution curves of DSRC-NCG with different SiO_2_ contents.

The multiscale pore-network architecture of DSRC-NCG was systematically characterized using SEM, AFM, nitrogen adsorption–desorption analysis, and three-dimensional μCT reconstruction to elucidate the structural evolution induced by SiO_2_ incorporation. The quantitative parameters extracted from these analyses are summarized in Table 1, Table 2, Table 3 and Table 4, with representative micrographs shown in Figure 1 and Figure 2.

At the microscale, SEM observations reveal that DSRC-NCG forms an irregular sponge-like framework after freeze-drying, accompanied by local shrinkage-induced cracking. Large pores are primarily distributed within the 20–80 μm range (Figure 1a). At higher magnification, a honeycomb-like porous network with pore sizes concentrated at 0.2–2.0 μm and wall thicknesses of approximately 70–180 nm is observed, indicating a partially interconnected skeleton. High-resolution SEM images (Figure 1b) further show that SiO_2_ nanoparticles (60–120 nm) are uniformly embedded within the pore walls, with bright protrusions appearing at junctions, suggesting localized reinforcement arising from particle–polymer interactions.

AFM analysis provides complementary insight into nanoscale surface topography. The two-dimensional height maps (Figure 1c) exhibit granular features and shallow grooves, with arithmetic roughness (Ra) values increasing from 9.8 to 14.9 nm as the SiO_2_ content rises from 0.08 to 0.20 wt% (Table 1 and Table 2). Three-dimensional height maps (Figure 1d) reveal more pronounced protrusions and fine agglomerates, highlighting the formation of a composite “matrix–particle” interface where nanoscale SiO_2_ domains integrate with the polymer backbone.

Quantitative SEM/AFM analysis indicates that increasing SiO_2_ content from 0.08 to 0.20 wt% leads to continuous refinement of the network structure, manifested by reduced fiber diameters (*d*_f_), increased node densities (*ρ*_node_), and shortened mesh characteristic lengths (*ξ*). The network becomes most compact and uniform at 0.16–0.20 wt%, where *d*_f_ stabilizes at 78–83 nm and ρ_node reaches 1.98–2.15 μm^−2^ (Table 1). Beyond this range (≥0.22 wt%), partial nanoparticle aggregation induces slight coarsening, consistent with the observed increase in *ξ* and *D*c.

At the mesoscale, nitrogen adsorption–desorption analysis further confirms the structural optimization induced by moderate SiO_2_ loading. As shown in Table 3, the specific surface area increases from 112.4 to 152.9 m^2^/g and pore volume from 0.382 to 0.495 cm^3^/g as SiO_2_ content increases to 0.20 wt%, while the average pore size decreases from 13.6 to 11.2 nm. The pore-size distribution curves (Figure 2) evolve from a broad distribution at low SiO_2_ content toward a narrower and more uniform profile within the 0.16–0.20 wt% range, indicating effective pore refinement. At 0.24 wt%, both surface area and pore volume decrease and the distribution broadens again, reflecting aggregation-induced structural heterogeneity.

At the three-dimensional scale, μCT-based pore-network reconstruction reveals a consistent connectivity evolution trend (Table 4). As the SiO_2_ content increases from 0.08 to 0.20 wt%, the connectivity index rises from 0.46 to 0.67, the dominant-channel proportion increases from 34.2% to 51.9%, and the average channel length extends from 18.6 to 24.1 μm. These changes indicate a transition from a “discrete short-chain” pore network to a more continuous and efficient transport pathway. Further increasing the SiO_2_ content to 0.24 wt% leads to a pronounced decline in all connectivity metrics, attributed to nanoparticle aggregation causing local pore blockage and pathway interruption.

Overall, multiscale structural characterization consistently demonstrates that DSRC-NCG exhibits an optimal “fine-fiber, high-node-density, uniformly connected” network within the SiO_2_ content range of 0.16–0.20 wt%. This hierarchical structural optimization provides a robust physical basis for subsequent interfacial regulation and flow-response behavior.

#### 2.1.2. Surface Chemical Characteristics and Interfacial Wettability

**Figure 3:**
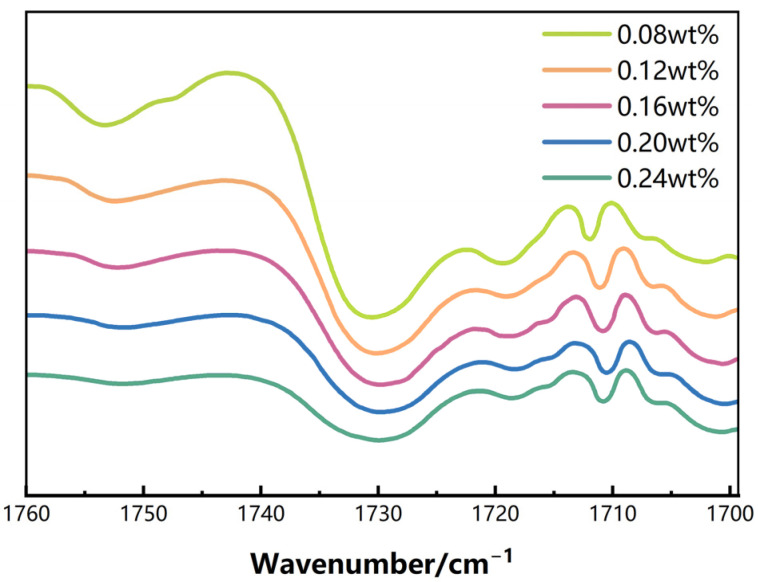
FTIR spectra of DSRC-NCG with different SiO_2_ contents in the C=O stretching vibration region (1760–1698 cm^−1^).

**Figure 4:**
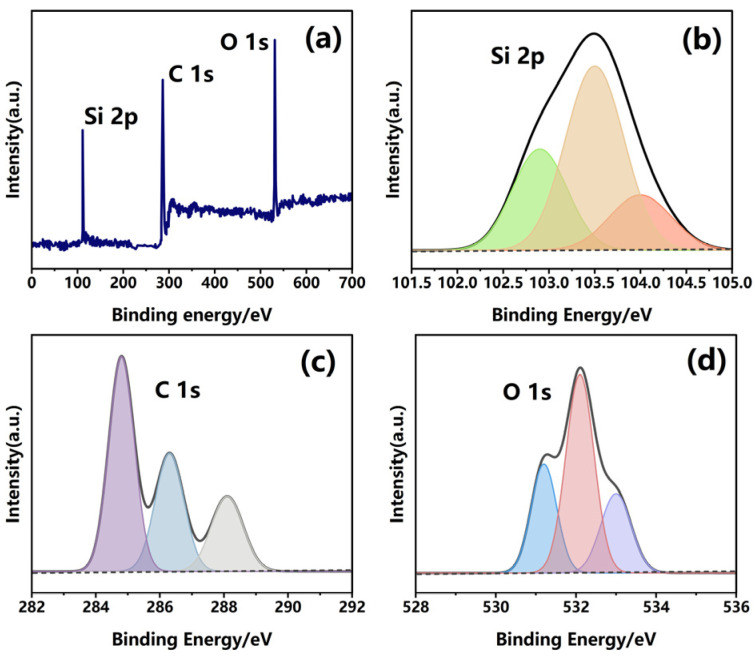
XPS characterization of DSRC-NCG at an SiO_2_ content of 0.20 wt% (representative sample): ((**a**) Survey scan; (**b**) Si 2p core-level spectrum; (**c**) C 1s core-level spectrum; (**d**) O 1s core-level spectrum). The different colored curves represent the fitted peak components corresponding to different chemical states in each core-level spectrum.

To elucidate the surface chemical composition and interfacial properties of DSRC-NCG, FTIR, XPS, and static contact-angle measurements were conducted on samples containing varying SiO_2_ contents. The results are summarized in Table 5 and Table 6 and Figure 3 and Figure 4.

FTIR spectra (Figure 3, Table 5) reveal that the C=O stretching vibration peak gradually shifts from 1731.4 cm^−1^ to 1726.1 cm^−1^ as the SiO_2_ content increases from 0.08 wt% to 0.20 wt%. Meanwhile, the intensity of the Si–O–Si absorption band increases from 0.84 to 1.24 (Table 2). These changes indicate enhanced dispersion of SiO_2_ within the polymer matrix and strengthening of polymer–particle interfacial interactions. When the filler content is further increased to 0.24 wt%, the C=O peak shifts back to 1727.5 cm^−1^ and the Si–O–Si intensity decreases to 1.10, suggesting that excessive nanoparticles lead to aggregation and a reduction in effective interfacial contact area.

XPS analysis of a representative DSRC-NCG sample with an SiO_2_ content of 0.20 wt% (Figure 4) corroborates these observations. With increasing SiO_2_ content, the O/C atomic ratio increases from 0.36 to 0.51, indicating progressive enrichment of oxygen-containing functional groups at the surface. The Si 2p peak area also increases consistently, implying enhanced exposure of SiO_2_ on the material surface. At 0.24 wt%, however, the O/C ratio decreases to 0.47, consistent with the aggregation-induced reduction in accessible polar groups observed in FTIR (Table 6).

Contact-angle measurements further demonstrate that incorporation of SiO_2_ modifies the surface wettability of DSRC-NCG from hydrophobic to increasingly hydrophilic, with the contact angle decreasing from 92.5° to 78.4° as the SiO_2_ content increases to 0.20 wt%. A subsequent rise to 83.2° at 0.24 wt% is again attributed to nanoparticle clustering and the associated decrease in exposed polar groups.

Overall, DSRC-NCG exhibits the most favorable surface chemical stability and hydrophilicity within the SiO_2_ content range of 0.16–0.20 wt%. At higher filler loadings, nanoparticle aggregation disrupts interfacial homogeneity and weakens these beneficial effects. These findings establish a solid basis for understanding the interfacial interactions between the gel and pore–throat structures in subsequent analyses.

#### 2.1.3. Rheological Behavior and Mechanical Stability

**Figure 5:**
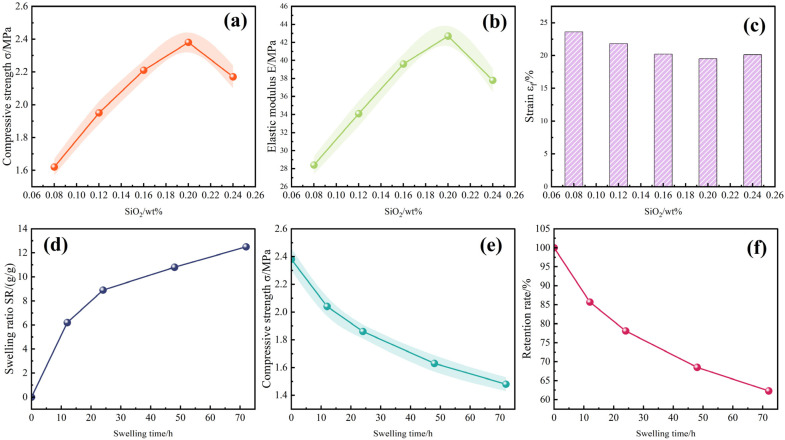
Mechanical properties and stability of DSRC-NCG under different SiO_2_ contents and swelling durations: ((**a**) compressive strength *σ* with SiO_2_ content; (**b**) elastic modulus E with SiO_2_ content; (**c**) failure strain *ε*_f_ as a function of SiO_2_ content; (**d**) swelling ratio SR at different swelling times (0.20 wt% SiO_2_); (**e**) compressive strength of swollen gels; (**f**) strength retention after swelling).

To comprehensively evaluate the rheological characteristics and mechanical stability of DSRC-NCG, rotational rheometry, DMA analysis, and compression–swelling coupling experiments were conducted on samples containing different SiO_2_ loadings. The corresponding results are summarized in Table 7 and Figure 5.

Rheological measurements show that both viscosity and storage modulus (G′) increase substantially as the SiO_2_ content rises from 0.08 wt% to 0.20 wt%. Specifically, viscosity increases from 12.4 Pa·s to 32.5 Pa·s, and G′ increases from 1860 Pa to 4120 Pa, while the G′/G″ ratio increases from 1.49 to 2.56 (Table 7). These trends indicate a progressive enhancement of the elastic contribution and a transition of the network structure from a “loosely connected” to a “reinforced” configuration. When the filler loading is further increased to 0.24 wt%, both viscosity and G′ decrease (29.1 Pa·s and 3650 Pa, respectively), accompanied by a drop in modulus retention from 91.7% to 85.2%, which is attributed to diminished load-transfer efficiency caused by nanoparticle aggregation.

Compression testing further validates these observations. As the SiO_2_ content increases from 0.08 wt% to 0.20 wt%, compressive strength increases from 1.62 MPa to 2.38 MPa, elastic modulus increases from 28.4 MPa to 42.7 MPa, and failure strain (*ε*_f_) decreases from 23.6% to 19.5% (Figure 5a–c). These results reflect a transition from a compliant to a densified network structure, consistent with the modulus-enhancing effect observed in the rheological tests. When the filler content is raised to 0.24 wt%, both strength and modulus decline, while *ε*_f_ slightly increases to 20.1%, indicating that aggregation creates structural discontinuities.

Swelling–mechanical coupling experiments reveal that the swelling ratio (SR) increases from 0 to 12.5 g/g over 72 h, whereas the mechanical response exhibits nonlinear degradation. During the rapid swelling stage (0–12 h), compressive strength decreases to 2.04 MPa, yet the strength retention remains above 85%, indicating that the network maintains considerable load-bearing capacity even while hydrating. At prolonged swelling durations (72 h), compressive strength decreases to 1.48 MPa (retention of 62.3%), reflecting network rearrangement and reduced toughness during long-term immersion (Figure 5d–f).

Overall, both the rheological and mechanical properties of DSRC-NCG exhibit a continuous “enhancement–optimization–degradation” evolution with increasing SiO_2_ content, with the optimal performance occurring at 0.16–0.20 wt%. Moderate SiO_2_ loadings promote network densification and strengthen interfacial bonding, significantly improving load-bearing capacity, elasticity, and environmental stability. Excessive filler content, however, leads to aggregation-induced heterogeneity, diminishing these enhancements.

From a field-injectivity perspective, the observed rheological evolution has direct implications for DSRC-NCG deployment under practical shear conditions. During surface pumping and near-wellbore injection, the gel experiences relatively high shear rates, under which the moderate viscosities measured at 10 s^−1^ (12.4–32.5 Pa·s) indicate that DSRC-NCG remains injectable without excessive pressure buildup. The progressive increase in G′/G″ with SiO_2_ content reflects enhanced elastic dominance, which is beneficial for post-injection structural integrity but does not preclude flow under shear-dominated injection regimes.

Importantly, the optimal SiO_2_ range (0.16–0.20 wt%) represents a balance between injectivity and in situ plugging efficiency. At this compositional window, the gel exhibits sufficient shear resistance to maintain network integrity after placement, while avoiding the excessive viscosity or structural heterogeneity that could impair injectivity. In contrast, higher SiO_2_ loading (0.24 wt%) leads to aggregation-induced heterogeneity, which may increase flow resistance under variable shear conditions and reduce effective injectivity in tight pore–throat systems. These results suggest that DSRC-NCG can achieve favorable “pumpability–placement–stability” coordination under realistic field shear environments, supporting its applicability for deep-water tight reservoir flow regulation.

Collectively, these structural, interfacial, and mechanical characteristics define the multiscale structural basis required for understanding the subsequent pore–flow coupling and flow-regulation mechanisms discussed in Section 2.3.

### 2.2. Pore-Throat Structure Regulation and Flow-Response Mechanisms

Building upon the structural characteristics identified in Section 2.1, this section systematically examines how pore–throat regulation by DSRC-NCG translates into macroscopic flow responses, thereby providing direct experimental evidence for the structure–flow coupling mechanism.

#### 2.2.1. Multiscale Flow Response Driven by Pore–Throat Structural Evolution

To assess the adaptability of DSRC-NCG across reservoir systems with different permeability levels, three multiscale flow configurations—microfluidic models (pore-scale pore–flow interaction), artificial cores (core-scale flow matching), and dry-gel-coated sandpacks (near-wellbore heterogeneity adaptation)—were established to systematically evaluate pore–flow matching and heterogeneity adaptation under increasing SiO_2_ content. The comprehensive flow response was quantified using dominant-channel fraction (*f*_dom_), pore–flow matching index (HCI), residual resistance factor (RRF), injectivity index (J), and pressure-decay behavior. The combined indicators consistently reveal a “progressive optimization–subsequent attenuation” pattern with increasing SiO_2_ content, reflecting the gel’s structure–flow coupling mechanism.

**Figure 6:**
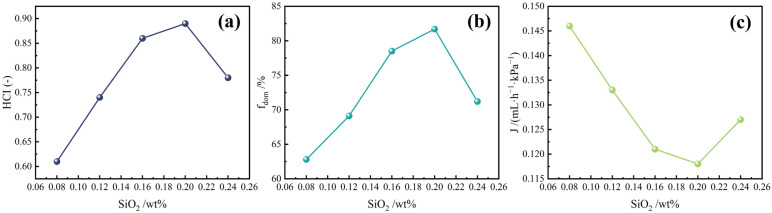
Pore–flow matching and channel reconstruction behavior of DSRC-NCG under different SiO_2_ contents (microfluidic model and composite indicators, n = 3). (**a**) hierarchical compatibility index (HCI) as a function of SiO_2_ content; (**b**) dominant flow ratio (f_dom_) as a function of SiO_2_ content; (**c**) injection flux (J) as a function of SiO_2_ content).

As shown in Figure 6, *f*_dom_ increases from 62.8% to 81.7% and HCI rises from 0.61 to 0.89 as the SiO_2_ content increases from 0.08 wt% to 0.20 wt%. The dominant transport pathways evolve from “coarse and highly connected” channels to “multi-branch, fine-scale” networks, indicating a more uniform flux distribution. The increase in *f*_dom_ confirms that the primary flow channels are effectively refined and subdivided, while the simultaneous improvement in HCI indicates that blocking efficiency in low-, medium-, and high-permeability zones becomes more balanced, representing optimal overall matching performance.

At 0.24 wt% SiO_2_, however, *f*_dom_ decreases to 71.2% and HCI drops to 0.78, suggesting that excessive nanoparticle loading induces aggregation, disrupts fine-pore connectivity, and increases bypass pathways, thereby reducing the system’s ability to regulate heterogeneous reservoirs uniformly.

**Figure 7:**
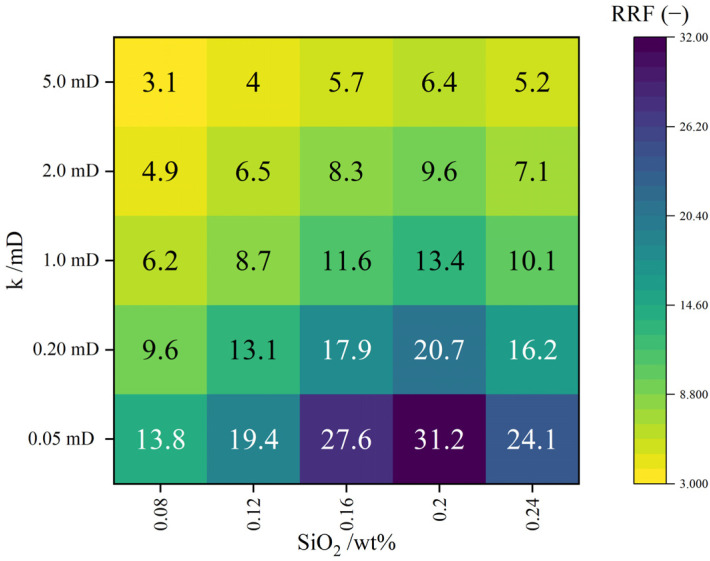
Heat map of the RRF–permeability (K) distribution for DSRC-NCG with different SiO_2_ contents.

The RRF–K heat map (Figure 7) further reveals a distinct two-dimensional distribution pattern. Along the SiO_2_-content direction, RRF increases across all permeability intervals and forms a continuous “high-response band” within the optimal window of 0.16–0.20 wt%. Along the permeability direction, RRF increases most significantly in the low-permeability region (0.05–0.20 mD), indicating that nanoparticles preferentially form denser networks within fine pores, enhancing flow resistance. In the medium-to-high permeability region (1.0–5.0 mD), RRF also increases but with a gentler gradient, reflecting the “refinement of coarse channels.”

At the optimal 0.20 wt% SiO_2_, the highest RRF values are obtained across all permeability levels (31.2 → 6.4 with increasing K), confirming strong cross-permeability control. At 0.24 wt%, however, a local “cold zone” reappears, with reduced RRF in low-permeability cores due to aggregation-induced pore blockage and bypass flow.

This two-dimensional pattern indicates that the 0.16–0.20 wt% interval is the optimal range for achieving “fine-pore connection, coarse-channel refinement, and cross-permeability balance,” consistent with the microfluidic observations.

**Figure 8:**
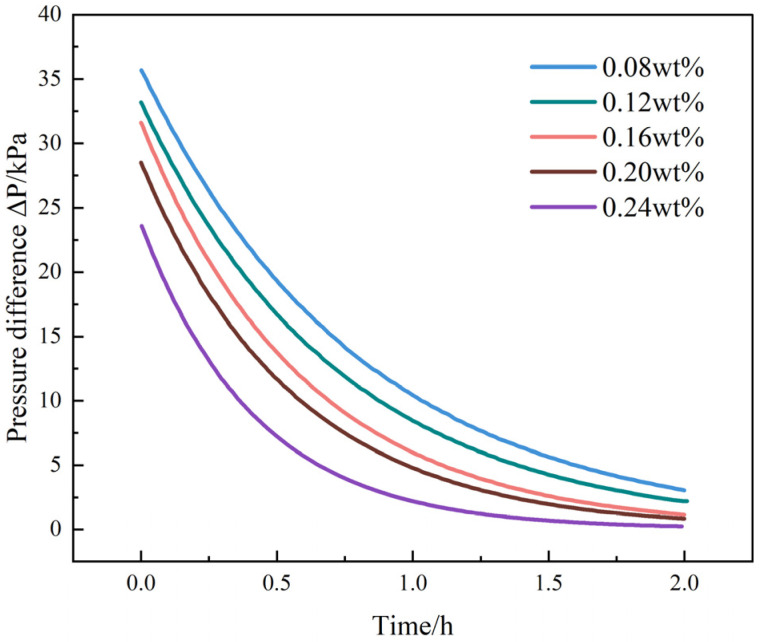
Pressure–time response curves of DSRC-NCG with different SiO_2_ contents under identical 1.0 mD core-flooding conditions.

Injectivity index (J) and pressure–time curves (Figure 8, Table 8) further confirm the “blocking–injectivity balance” within the optimal nanoparticle range. Both initial and steady-state J values decrease as SiO_2_ content increases, yet at 0.20 wt%, the system still maintains a practical injectivity window (steady-state J = 0.121 mL·h^−1^·kPa^−1^), indicating no injection difficulties. Meanwhile, the peak pressure reaches its maximum (35.7 kPa), and the half-decay time is the longest (0.81 h), reflecting strengthened gel network stability, reduced leak-off, and the highest blocking intensity.

At 0.24 wt%, although the peak pressure remains high, the sharper pressure decay (0.60 h) suggests “localized plugging + bypass flow,” consistent with the reductions in *f*_dom_ and HCI.

**Figure 9:**
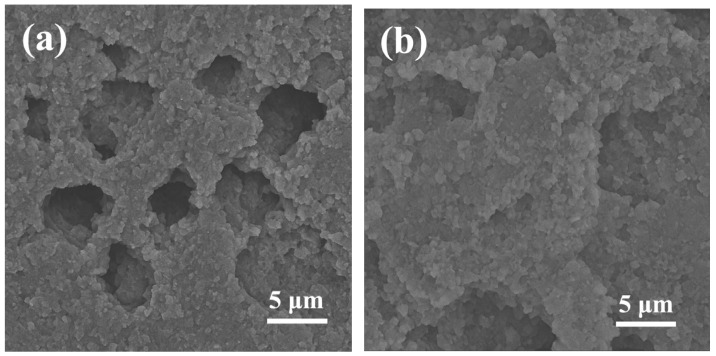
SEM images of tight sandstone samples before and after gel treatment ((**a**) before treatment, (**b**) after treatment).

**Figure 10:**
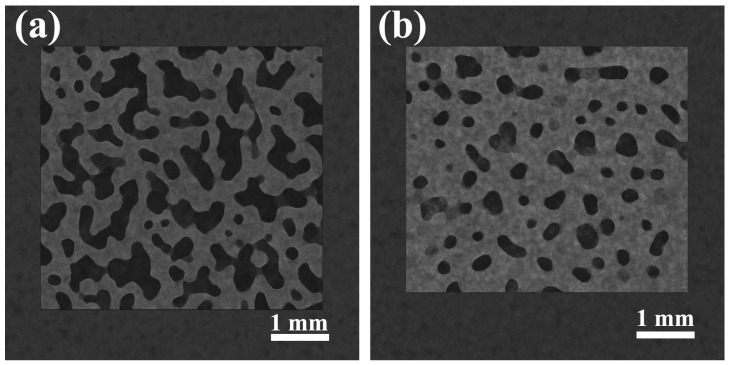
Micro-CT cross-sectional images of tight sandstone samples before and after gel treatment ((**a**) before treatment, (**b**) after treatment).

To directly link flow behavior with structural evolution, SEM and micro-CT observations before and after gel treatment are shown in Figure 9 and Figure 10. At the microscale, gel treatment smooths grain surfaces, fills or bridges pore throats, and reduces pore-edge roughness. At the mesoscale, micro-CT images reveal partial occupation and disruption of dominant flow channels, resulting in reduced pore connectivity and more homogeneous grayscale distributions. These multiscale morphological changes provide direct structural evidence for the observed redistribution of flow pathways and enhancement of pore–flow matching.

These multiscale flow responses collectively indicate that DSRC-NCG does not simply block pores, but actively reconstructs dominant flow pathways through structure–flow coupling, which is further integrated and conceptualized in Section 2.3.

#### 2.2.2. Quantitative Structural Regulation, Network Evolution, and Reversibility

Under simulated deep-water tight-reservoir conditions of 90 °C, 35 MPa, and a salinity of 30.0 g/L, in situ micro-CT sequential imaging revealed a typical three-stage structural evolution during the injection of DSRC-NCG. As the injected pore volume increased from 0.3 PV to 0.6 PV, the connected porosity *φ*_c_ increased from 12.1% to 12.9%, the dominant-channel volume fraction *V*_dom_ increased from 9.4% to 10.7%, the coordination number Z rose from 2.71 to 2.96, and the tortuosity *τ* decreased from 1.63 to 1.55 (Table 9). These changes indicate preferential occupation of dominant channels by the gel, driving the pore network toward a state characterized by high connectivity and low tortuosity.

During the subsequent gel-setting stage (2 h), the structural parameters were further optimized, with φ_c_ reaching 13.1%, *V*_dom_ 11.0%, Z 3.04, and *τ* 1.52. Correspondingly, the permeability values obtained from LBM inversion and steady-state testing decreased to 0.20 mD and 0.19 mD, respectively. This suggests selective filling of dominant channels and the formation of stable localized blockage zones. During chemical degradation, the experimentally measured permeability *k*_exp_ increased to 0.35 mD at 12 h and further to 0.43 mD at 24 h, while *φ*_c_ and *τ* partially returned toward their initial values (12.5%, 1.58 at 12 h; 11.8%, 1.66 at 24 h). This reflects a reversible flow-response behavior consisting of blockage followed by partial reopening.

The structural parameters obtained under different SiO_2_ contents further demonstrate a clear composition-window effect (Table 10). Within the range of 0.16 to 0.20 wt%, *V*_dom_ reached its highest values (11.5–11.8%), *τ* reached its lowest values (1.51–1.49), and *k*_exp_ reached its minimum values (0.22–0.19 mD). This indicates that the gel network in this range can simultaneously enhance channel coordination, reduce flow-path tortuosity, and effectively suppress bypass flow. In contrast, when SiO_2_ content exceeded 0.24 wt%, particle aggregation reduced throat radius rth, caused *χ* to decrease sharply (more negative), and resulted in the passivation of dominant channels, thereby increasing *k*_exp_ (0.27–0.38 mD).

Overall, the pore–throat network parameters (*φ*_c_, *V*_dom_, *Z*, *τ*, *χ*) and flow properties (*k*_img_, *k*_exp_) exhibit synchronized evolution and strong composition dependence. This forms a continuous response process that includes channel restructuring, localized blockage, and partial reopening, supporting the coupled mechanism by which DSRC-NCG regulates seepage behavior through structural modification.

Following gel degradation, both micron- and nanometer-scale parameters recover toward their original states, with recovery ratios exceeding 90%, demonstrating that the structural regulation induced by DSRC-NCG is predominantly reversible. Based on the combined evaluation of throat contraction, distribution convergence, and surface-area enhancement, the structural regulation index (SRI) reaches its highest values at 0.16–0.20 wt%, confirming this compositional window as optimal for achieving stable, balanced, and reversible pore–throat regulation.

Overall, the coupled evolution of pore–throat structure and flow response reveals a continuous mechanism involving dominant-channel refinement, selective blockage, and partial reopening. This structure–flow coupling explains the observed balance between strong conformance control and maintained injectivity, providing a mechanistic foundation for the application of DSRC-NCG in tight-reservoir flow regulation.

At the micron scale, the dominant throat radius decreases progressively from the untreated value of 0.353 µm to 0.308, 0.271, and 0.248 µm, corresponding to reductions of 12.7%, 23.2%, and 29.7%, respectively. Meanwhile, the distribution width (FWHM) narrows from 0.212 µm to 0.187, 0.171, and 0.163 µm, indicating an evolution from dispersed to concentrated throat dimensions. Within this concentration range, the bimodality index remains stable at 0.09–0.10, with no indication of structural divergence. By contrast, at 0.24 wt%, the emergence of two dominant throat radii (0.236 µm and 0.381 µm), along with the increase in FWHM to 0.272 µm and BI to 0.34, demonstrates clear non-uniformity introduced by high gel concentration (Table 11).

At the nanometer scale, mesopore structure evolution mirrors the micron-scale trend. The BJH dominant peak decreases from 11.82 nm to 10.73, 9.86, and 9.62 nm; the distribution width narrows from 5.62 nm to 5.27, 4.81, and 4.56 nm; and the specific surface area increases from 7.42 m^2^/g to 8.15, 8.94, and 9.18 m^2^/g, corresponding to increases of 9.8 percent, 20.5 percent, and 23.7%, respectively. However, at 0.24 wt%, dual mesopore peaks (9.47 nm and 12.21 nm) reappear, the distribution width increases to 6.13 nm, and the specific surface area decreases to 9.02 m^2^/g, consistent with the non-uniform behavior observed at the micron scale.

Following gel degradation, the pore–throat structure exhibits a clear reversible recovery. The dominant throat radius returns to 0.331 µm, equivalent to 93.8% of the untreated sample. The BJH dominant peak returns to 11.33 nm (95.8% recovery), and the specific surface area decreases to 7.88 m^2^/g (106% recovery). This recovery indicates that the structural regulation induced by the gel is primarily governed by reversible adhesion, filling, and interface-modification mechanisms, rather than irreversible damage to the mineral framework.

Based on the combined evaluation of throat contraction, distribution convergence, and surface-area enhancement, the structural regulation index (SRI) demonstrates that the 0.16 wt% and 0.20 wt% samples achieve the highest values of 0.29 and 0.33, reflecting the most effective structural adjustment. In contrast, the SRI of the 0.24 wt% sample decreases to 0.05, consistent with the bimodal patterns observed at both scales. Collectively, these findings indicate that nanocomposite gel at moderate concentrations (0.16–0.20 wt%) can achieve simultaneous throat refinement, distribution convergence, and surface-area gain, whereas excessive concentrations lead to particle aggregation, reduced structural uniformity, and diminished stability of flow pathways.

### 2.3. Field-Scale Implications for Improved Oil Recovery

From a reservoir engineering perspective, the multiscale pore–flow regulation behavior of DSRC-NCG observed in this study has direct implications for improved oil recovery in deep-water tight reservoirs. The refinement and redistribution of dominant flow channels, as evidenced by increased HCI and optimized RRF distributions, are expected to enhance sweep efficiency by mitigating early breakthrough through high-permeability streaks while maintaining connectivity within low-permeability zones. This balanced cross-permeability control is particularly critical for tight reservoirs, where production is often governed by a limited number of preferential flow paths.

At the field scale, such pore–flow matching behavior translates into more uniform displacement fronts during waterflooding or gas injection, delayed water cut rise, and improved utilization of previously unswept reservoir volumes. The identification of an optimal SiO_2_ content window (0.16–0.20 wt%) further suggests that DSRC-NCG can be designed to achieve effective conformance control without compromising injectivity, thereby supporting sustained production and incremental oil recovery. While the present study focuses on mechanistic and multiscale experimental evaluation, the established structure–flow coupling framework provides a quantitative basis for subsequent reservoir-scale simulation and pilot implementation.

### 2.4. Coupled Mechanism of Flow Behavior

**Figure 11:**
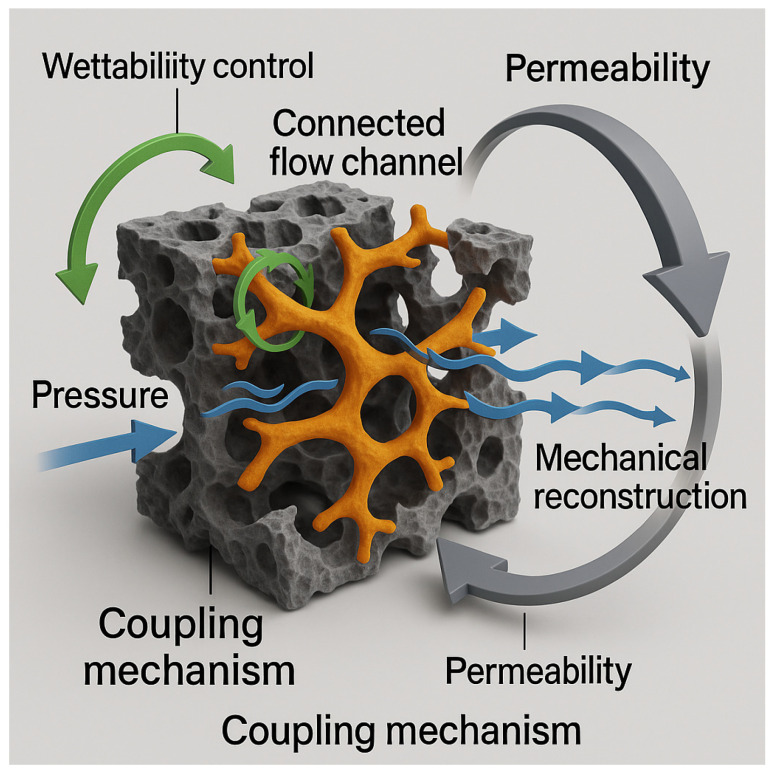
Schematic illustration of the seepage coupling mechanism of DSRC-NCG in porous media, highlighting the interplay of pore structure, capillary force, and viscous resistance in dynamic channel evolution (Orange regions denote reconstructed dominant flow channels, blue arrows indicate fluid flow, green arrows represent wettability regulation, and gray circular arrows illustrate permeability–mechanical reconstruction feedback, highlighting dynamic channel evolution).

The abbreviations used in this study are summarized in Appendix A. Based on the multi-scale structural descriptors (including *r*_mode_, FWHM, *Z*, *τ*, and *V*_dom_) and the flow-response indicators (such as *f*_dom_, HCI, RRF, and J), the flow behavior of DSRC-NCG in deepwater tight reservoirs can be interpreted as a continuous coupling process consisting of pore-throat network reconstruction, flow-path redistribution, and reversible flow regulation (Figure 11).

First, pore-throat restructuring provides the fundamental basis for flow evolution. Within the optimal concentration window of 0.16–0.20 wt%, the MICP–BET results show that the dominant throat size decreases from 0.353 μm to 0.248 μm, while the specific surface area increases to 9.18 m^2^/g. Correspondingly, the μCT-derived connectivity *Z* increases from 2.42 to 3.04, tortuosity *τ* decreases from 1.78 to 1.52, and the dominant-channel volume fraction *V*_dom_ increases from 7.6% to 11.0%. These coordinated changes indicate that the gel produces a combined “refinement–bridging” effect across the micro–nano scales, transforming a network previously governed by a few coarse channels into a more uniform and low-tortuosity flow pathway.

Second, the structural restructuring directly drives selective flow-path regulation. Microfluidic and core-flooding experiments show that *f*_dom_ increases from 62.8% to 81.7% within the optimal concentration range, while HCI rises from 0.61 to 0.89. The RRF values for the low-, medium-, and high-permeability cores simultaneously increase and form a continuous high-response zone, demonstrating that coarse channels are effectively refined and fine channels are reconnected. Although the injectivity index J decreases with increasing concentration, it remains within an acceptable injection window at 0.20 wt% (steady J = 0.121 mL·h^−1^·kPa^−1^), indicating a balanced state where sealing strength is enhanced without compromising injectivity.

When the SiO_2_ content reaches or exceeds 0.24 wt%, the structural–flow coupling relationship reverses. μCT reveals the reappearance of peak splitting and an increase in *τ*, while *f*_dom_ and HCI decline and RRF exhibits cold zones. These phenomena indicate localized plugging accompanied by enhanced bypass flow, confirming that particle agglomeration disrupts the uniform regulation capability of the network.

Finally, during the degradation stage, both structural parameters and flow indicators show substantial recoverability. For example, *k*_exp_ rises from 0.19 mD to 0.43 mD, demonstrating that the gel primarily regulates flow through reversible adhesion and bridging effects rather than permanent pore-structure damage.

Overall, the coupled mechanism of DSRC-NCG can be summarized as follows: appropriate SiO_2_ content enables a refinement- and bridging-driven restructuring of the pore-throat network, guiding the flow paths toward uniformity and achieving cross-permeability collaborative control. Excessive SiO_2_ disrupts this coupling due to particle agglomeration. This mechanism is consistently supported by both structural parameters and flow-response functions.

## 3. Conclusions

(1) A narrow and operational compositional window (SiO_2_ = 0.16–0.20 wt%) is identified as the design rule for DSRC-NCG under deep-water tight-reservoir conditions. In this window, the gel simultaneously maximizes (i) network continuity and stability, (ii) surface polarity/wettability, and (iii) rheological/mechanical robustness, whereas over-loading (≥0.24 wt%) triggers aggregation-driven heterogeneity that systematically degrades these coupled properties.

(2) Cross-scale evidence establishes a quantitative “structure → pore–throat → flow” linkage for DSRC-NCG. Moderate SiO_2_ loading produces coordinated throat refinement and distribution convergence (micro–nano pore metrics), which translates into dominant-channel redistribution and improved cross-permeability conformance (*f*_dom_, HCI, RRF), while maintaining a practical injectivity window (J). This verifies that the optimal formulation achieves “plugging strength–injectivity coordination”, rather than pursuing plugging at the expense of placement.

(3) A reversible structure–flow coupling mechanism is confirmed and can be summarized as selective adsorption/filling → dominant-channel refinement and stabilization → chemically triggered partial reopening. Time-resolved μCT and permeability responses show synchronized evolution of connectivity/topology (*ϕ*_c_, *V*_dom_, *Z*, *τ*, *χ*) with (*k*_img_, *k*_exp_), demonstrating that regulation is dominated by reversible interfacial adhesion and occupancy instead of irreversible pore damage. This mechanism provides the central rationale for low-damage, controllable conformance control in tight pore–throat networks.

(4) Field-scale implications: the identified window (0.16–0.20 wt% SiO_2_) offers a deployable balance among placement (injectivity), in situ conformance (cross-permeability control), and post-treatment reversibility, which is essential for improved sweep efficiency in heterogeneous tight reservoirs. The formulation and pumping workflow are compatible with conventional operations, supporting scalability. Key implementation challenges are expected to be dominated by reservoir-scale heterogeneity, long-term thermal exposure, and injection–production coupling that may affect placement uniformity and degradation controllability. Accordingly, future work should prioritize long-duration coreflooding, large-scale physical modeling, and pilot validation to quantify placement efficiency, longevity, and controllable reopening under representative field boundary conditions.

## 4. Materials and Methods

### 4.1. Experimental Materials

Acrylamide (AM, purity ≥ 99.0%, Aladdin Reagent Co., Ltd., Shanghai, China); 2-acrylamido-2-methylpropane sulfonic acid (AMPS, purity ≥ 98.0%, Aladdin Reagent Co., Ltd., Shanghai, China); amine-functionalized silica nanoparticles (NH_2_–SiO_2_, average particle size 60–120 nm, purity 99.8%, Nanjing XFNANO Materials Tech Co., Ltd., Nanjing, China); β-cyclodextrin-grafted polymer solution (solid content 20%, Shanghai Macklin Biochemical Co., Ltd., Shanghai, China); adamantane-grafted polymer solution (solid content 20%, Shanghai Macklin Biochemical Co., Ltd., Shanghai, China); sodium chloride (NaCl, purity ≥ 99.5%, Sinopharm Chemical Reagent Co., Ltd., Shanghai, China); calcium chloride dihydrate (CaCl_2_·2H_2_O, purity ≥ 99.0%, Sinopharm Chemical Reagent Co., Ltd., Shanghai, China); sodium hydroxide (NaOH, purity ≥ 96.0%, Sinopharm Chemical Reagent Co., Ltd., Shanghai, China); artificial tight sandstone cores (gas permeability 0.05–5.0 mD, Haian Petroleum Scientific Instrument Co., Ltd., Jiangsu, China); and glass microfluidic chips (channel width 0.2–8.0 µm, Wuxi NEMS Microfluidics Technology Co., Ltd., Wuxi, China). The composition of the DSRC-NCG gel system and the functional roles of its key components are summarized in Table 12.

### 4.2. Experimental Instruments

BS-210S electronic balance (Beijing Sartorius Scientific Instruments Co., Ltd., Beijing, China); TDL-5-A benchtop low-speed centrifuge (Shanghai Anting Scientific Instrument Co., Ltd., Shanghai, China); JJ-1 precision electric stirrer (Changzhou Guohua Electric Appliance Co., Ltd., Changzhou, China); LGJ-10 vacuum freeze dryer (Beijing Songyuan Huaxing Technology Development Co., Ltd., Beijing, China); FTIR-650 Fourier-transform infrared spectrometer (Tianjin Gangdong Technology Development Co., Ltd., Tianjin, China); KYKY-EM6900 scanning electron microscope (Beijing Zhongke Instrument Co., Ltd., Beijing, China); SBC-12 ion sputter coater (Beijing Zhongke Instrument Co., Ltd., Beijing, China); JW-BK200 automated specific-surface-area and pore-size analyzer (Beijing JWGB Sci & Tech Co., Ltd., Beijing, China); JC2000D contact-angle goniometer (Shanghai Zhongchen Digital Technology Equipment Co., Ltd., Shanghai, China); WDW-100 microcomputer-controlled universal testing machine (Jinan Shijin Group Co., Ltd., Jinan, China); RS-1500 high-temperature/high-pressure rheometer (Shanghai Baosheng Industrial Development Co., Ltd., Shanghai, China); nanoVoxel-2000 microfocus X-ray CT scanner (Tianjin Sanying Precision Instruments Co., Ltd., Tianjin, China); HTHP-IV multifunctional core-flooding system (Jiangsu Hai’an Petroleum Scientific Instrument Co., Ltd., Nantong, China); XDS-3 inverted biological microscope (Chongqing Optical Instrument Co., Ltd., Chongqing, China). AutoPore V 9620 mercury intrusion porosimeter (Micromeritics Instrument Corp., Norcross, GA, USA); ASAP 2460 nitrogen adsorption–desorption analyzer (Micromeritics Instrument Corp., Norcross, GA, USA). Image processing was performed using ImageJ (v1.53); AFM data were analyzed using NanoScope Analysis (v1.9); and data processing and plotting were carried out using MATLAB (v2024).

### 4.3. Synthesis Procedure

**Figure 12:**
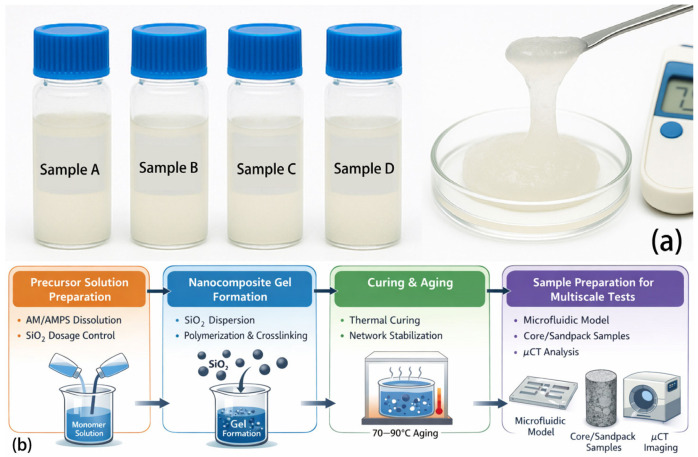
(**a**) Appearance and macroscopic morphology of DSRC-NCG nanocomposite gel samples with different SiO_2_ contents; (**b**) Schematic flowchart illustrating the synthesis and preparation procedure of DSRC-NCG.

In a 500 mL glass beaker, 180.0 mL of deionized water was added and maintained at 35 °C under magnetic stirring at 350 r/min for 5 min. A total of 1.20 g of acrylamide/2-acrylamido-2-methylpropane sulfonic acid (AM/AMPS) copolymer powder (main-chain solid content 0.60 wt%) was slowly introduced in three portions at 3–5 min intervals, followed by continuous stirring for 40 min until the solution became clear and transparent. Separately, 0.35 g of amine-functionalized silica nanoparticles (NH_2_–SiO_2_, target concentration 0.175 wt%, average particle size 60–120 nm) was dispersed in 20.0 mL of deionized water and ultrasonicated using a probe sonicator at 100 W under an ice bath for 15–20 min. The resulting dispersion was added dropwise to the main solution over 5–10 min, followed by an additional 15 min of stirring. Subsequently, 1.3 mL of 15.0 wt% β-cyclodextrin-grafted polymer solution and 1.3 mL of 15.0 wt% adamantane-grafted polymer solution (molar ratio of active groups β-CD:Ad ≈ 1.1:1; soft crosslinking subnet accounting for 20% of the total solid content) were accurately added while stirring for 10 min. A high-salinity solution was prepared by dissolving 6.0 g of NaCl and 0.60 g of CaCl_2_·2H_2_O in 20.0 mL of deionized water. This salt solution was slowly introduced into the system under vigorous stirring to ensure a final equivalent salinity of 30.0 g/L. The pH was adjusted to 7.4–7.6 using 1.0 mol/L NaOH, followed by 3–5 min of aging. The mixture was then aliquoted into capped glass vials (20–30 mL each) and placed in a 70 °C thermostatic water bath for 90 min to allow the gel network to reach thermodynamic equilibrium and aging under reservoir-simulated temperature. After cooling to room temperature, the samples were subjected to a unified shear history (vortex mixer at 1 000 r/min for 2 min, or rheometer shear rate of 100 s^−1^ for 2 min) and allowed to stand for 60 min to obtain the standardized state prior to testing. The resulting material was the nanocomposite gel DSRC-NCG. To investigate the effect of SiO_2_ content on the structure and flow-regulation behavior of DSRC-NCG, a series of gel samples with different SiO_2_ concentrations were prepared by systematically adjusting the dosage of amine-functionalized silica nanoparticles (NH_2_–SiO_2_). Specifically, the SiO_2_ content was varied in the range of 0.08–0.28 wt% (relative to the total gel formulation), while all other parameters—including monomer concentration, crosslinking agent content, salinity, pH, temperature, stirring conditions, and curing protocol—were kept strictly constant. For each formulation, the synthesis procedure followed the same steps described above, differing only in the amount of NH_2_–SiO_2_ added. After gelation and curing, the resulting DSRC-NCG samples were labeled according to their corresponding SiO_2_ contents and subsequently used for microstructural characterization, pore-connectivity analysis, and flow experiments. The overall synthesis and preparation procedure is schematically summarized in Figure 12b to provide a step-by-step overview. For clarity and reproducibility, the overall synthesis and sample-preparation workflow is summarized as a step-by-step schematic in Figure 12b (together with the representative macroscopic appearance shown in Figure 12a).

### 4.4. Characterization Methods

#### 4.4.1. Scanning Electron Microscopy Characterization

To characterize the multiscale pore architecture of DSRC-NCG, samples were freeze-dried to preserve their native network morphology and subsequently coated with a 5–8 nm Au layer using an ion sputter coater to enhance conductivity. SEM imaging was performed using a Hitachi SU-8020 scanning electron microscope (Hitachi High-Technologies Corporation, Tokyo, Japan) at an acceleration voltage of 5 kV to visualize structural features across macro- and microscale levels.

The obtained images were analyzed with ImageJ (v1.53). The average fiber diameter (*d*_f_) was calculated from 50 randomly measured fibers in binarized images. Node density (*ρ*_node_) was determined by skeletonizing the network and counting the number of junctions within a 10 μm × 10 μm region, normalized to area density. Mesh characteristic length (*ξ*) was extracted from the mean equivalent diameter of pores in the skeleton–pore map. Cluster size (Dc) was obtained through connected-domain analysis.

#### 4.4.2. Atomic Force Microscopy Characterization

Surface features of DSRC-NCG were further examined using a Bruker Dimension Icon atomic force microscope (Bruker Corporation, Santa Barbara, CA, USA) in tapping mode. Freeze-dried samples were pressed into thin disks and mounted on mica substrates to ensure scanning stability. AFM imaging was conducted over 5 μm × 5 μm for both 2D and 3D height maps, with a scan rate of 0.5 Hz and a sampling resolution of 512 × 512 pixels.

AFM images were processed using NanoScope Analysis (v1.9), and the arithmetic average roughness (Ra) was directly extracted. Each dataset was evaluated from at least five images to ensure representativeness and reliability.

#### 4.4.3. FTIR and XPS Analyses

The surface chemical composition and interfacial characteristics of DSRC-NCG were examined using Fourier-transform infrared spectroscopy (FTIR) and X-ray photoelectron spectroscopy (XPS). FTIR spectra were collected using the KBr pellet method across 4000–400 cm^−1^ with a resolution of 4 cm^−1^. XPS measurements were acquired over 0–1200 eV using monochromated Al Kα radiation (1486.6 eV) with a pass energy of 0.1 eV, and peak positions were calibrated to the C 1s peak at 284.8 eV.

Interfacial wettability was assessed using a contact-angle goniometer. A 5 μL droplet of distilled water was deposited on the dried sample surface at room temperature, and the static contact angle was recorded as the average of three measurements.

### 4.5. Multiscale Evaluation Methods for Structure–Flow Regulation

#### 4.5.1. Mechanical Properties and Stability Tests

(1) Sample Preparation

DSRC-NCG samples, immediately after gelation, were poured into PTFE molds (diameter 20 mm, height 20 mm) and cured at 70 °C and 30.0 g/L salinity for 1.0 h. After curing, samples were demolded and trimmed into smooth cylinders with dimensional deviations ≤ 0.2 mm.

(2) Compression Mechanical Tests

Compressive properties were measured using an Instron 3367 universal testing machine. The loading rate was set to 1 mm/min, and samples were compressed until failure or until strain exceeded 50%. Stress–strain curves were recorded to determine the maximum compressive strength (*σ*_max_, MPa), failure strain (*ε*_f_, %), and modulus (*E*, MPa).

(3) Swelling Experiments

Swelling behavior was evaluated by immersing DSRC-NCG samples in a 70 °C, 30.0 g/L saline solution. Sample mass was recorded at 0, 3, 6, 12, 24, 36, and 48 h, and the swelling ratio was calculated as:

(1) $$SR=\frac{W_{t}-W_{0}}{W_{0}}$$

where *W*_t_ is the wet mass at time *t* (g), and *W*_0_ is the initial dry mass (g).

(4) Swelling–Mechanical Coupling Tests

At each swelling time point, samples were removed, blotted to remove surface water, and subjected to compression testing. The strength retention was calculated as:

(2) $$\sigma_{R}=\frac{\sigma_{t}}{\sigma_{0}}\times 100\%$$

where *σ*_t_ is the compressive strength at time *t* (MPa), and *σ*_0_ is the initial strength (MPa).

#### 4.5.2. Pore Connectivity and Dominant Pathway Characterization

DSRC-NCG samples with varying SiO_2_ contents were vacuum-dried at 60 °C for 24 h, and then cut into disks (diameter 5 mm, thickness 2 mm). Three-dimensional pore-structure imaging was performed using a SkyScan 1176 μCT system at a resolution of 9 μm.

Image reconstruction, threshold segmentation, and skeletonization were carried out using Avizo 9.0 to extract the 3D pore–throat network. Connectivity index (CI), average channel length, and dominant-flow-channel ratio were calculated using built-in modules. Each test was repeated three times, and results were averaged with an error margin within ± 5%.

#### 4.5.3. Multiscale Permeability Adaptability and Matching Experiments

Microfluidic chips (channel width: 0.2–8.0 μm; thickness: 50 μm) and artificial sandstone cores (diameter: 25 mm; length: 50 mm; permeability K = 0.05, 0.20, 1.0, 2.0, and 5.0 mD) were dried at 105 °C for 12 h prior to testing. The displacement system was maintained at 70 °C with a back pressure of 1.0 MPa. DSRC-NCG solutions with concentrations of 0.08–0.24 wt% were injected at 0.20 mL/h to a total volume of 0.30 PV, followed by a 12 h shut-in for gel network development. Subsequently, 2.0 PV of synthetic brine (35 g/L NaCl) was injected for post-flush.

The pressure drop across the cores was monitored in real time using a differential pressure sensor (resolution: 0.1 kPa). For microfluidic visualization, a CCD-equipped inverted microscope (30 fps) was used to capture flow field evolution. Images were processed by threshold segmentation to obtain the channel skeleton, and the dominant-flow-channel fraction (*f*_dom_) and channel connectivity were quantified.

Core permeability was calculated using Darcy’s law, and the residual resistance factor (RRF) was determined as:

(3) $$RRF=\frac{k_{before}}{k_{after}}$$

The plugging efficiency in each permeability zone was expressed as:

(4) $$\eta_{p}=1-\left(\frac{q_{after}}{q_{before}}\right)$$

To evaluate the overall pore–flow matching capability, the hierarchical compatibility index (HCI) was used. Weighting factors for low-, mid-, and high-permeability zones were set to w_L_:w_M_:w_H_ = 0.4:0.4:0.2, which can be adjusted based on the reservoir heterogeneity. Each measurement was repeated three times (n = 3), and mean values with standard deviations ≤ ±5% were reported. The HCI is defined as:

(5) $$\mathrm{HCI}=\frac{w_{L}\cdot \eta_{p,L}+w_{M}\cdot \eta_{p,M}+w_{H}\cdot \eta_{p,H}}{|\bar{\eta_{p}}-\eta_{p,L}|+|\bar{\eta_{p}}-\eta_{p,H}|+\varepsilon }$$

where L/M/H represent the low-, medium-, and high-permeability regions, $w_{i}$ is the area (or volume) fraction of each region, and $\bar{\eta_{p}}$ is the average plugging efficiency of the corresponding region. A small constant ε = 1 is applied to avoid division by zero. A larger HCI indicates stronger overall plugging and better uniformity among heterogeneous permeability domains.

#### 4.5.4. In Situ μCT Time-Sequence Scanning and Pore–Throat Evolution

A tight sandstone core (diameter 2.5 cm, height 5.0 cm) was mounted in a core holder with a confining pressure of 35 MPa and a back pressure of 10 MPa, under simulated deep-water conditions (90 °C, salinity 30.0 g/L).

After baseline scanning, DSRC-NCG (SiO_2_ = 0.20 wt%) was injected at 0.5 mL/min. Fast μCT scans were performed at 0.3 PV and 0.6 PV injection stages, followed by 2.0 h of static curing and additional scanning. The degradation stage was conducted using a γ-valerolactone/p-toluenesulfonic acid system (pH ≈ 2.5) for 12 h and 24 h, with corresponding scans.

Scanning was performed at 1.5–3.0 μm voxel resolution. This resolution range was selected to ensure adequate discrimination of pore–throat boundaries, dominant-channel connectivity, and local bridging or filling features induced by gel injection, while remaining compatible with the representative throat-size distribution (submicron to several microns) of the investigated tight sandstone. Preliminary tests confirmed that this resolution was sufficient to capture connectivity evolution without introducing excessive image noise or segmentation uncertainty. Grayscale thresholding and connected-domain filtering were used to extract pore and matrix phases. Skeletonization yielded key structural parameters including connected porosity $\phi_{c}$, dominant-channel volume fraction *V*_dom_, average throat radius rth, coordination number *Z*, tortuosity *τ*, and Euler characteristic *χ*.

For each scanning stage, the acquisition time was optimized to achieve stable grayscale contrast and an adequate signal-to-noise ratio required for reliable reconstruction, threshold segmentation, and skeleton-based network extraction, while avoiding unnecessary beam exposure or data redundancy. The same scanning protocol (voxel size, exposure settings, and reconstruction parameters) was applied throughout all stages to ensure consistency and comparability among time-sequence datasets.

To quantify the relationship between pore–throat structure and seepage capability, lattice Boltzmann simulations were performed to compute image-based permeability *k*_img_, which was compared with steady-state experimental permeability *k*_exp_. All scanning and flow processes were performed on the same core under identical confining and temperature conditions to ensure reproducibility and parameter closure.

#### 4.5.5. Pore–Throat Structure Characterization and Quantitative Analysis

Pore–throat structure was characterized using combined mercury intrusion porosimetry (MICP) and nitrogen adsorption–desorption (BET–BJH). Tight sandstone cores were obtained from representative deep-water formations and prepared as Φ25 mm × 25 mm cylinders for MICP. Portions were crushed to 60–80 mesh for nitrogen adsorption. All samples were vacuum-dried at 60 °C for 12 h before testing.

Nanocomposite gels with 0.12, 0.16, 0.20, and 0.28 wt% SiO_2_ were injected into cores at 1.5 MPa back pressure and 60 °C to 1.0 PV, followed by 12 h aging. Some samples were further degraded to assess reversibility.

MICP was conducted within 0.01–200 MPa to obtain cumulative intrusion curves. Throat diameters were calculated using the Washburn equation, and the logarithmic pore-size probability density function was used to extract modal throat radius r_mode_, full width at half maximum (FWHM), and bimodality index (BI). Nitrogen adsorption was performed at 77 K after 120 °C degassing for 8 h. BET specific surface area (S_BET_) was obtained from P/P_0_ = 0.05–0.30, and mesopore peak radius r_meso,mode_ and distribution width were derived using BJH analysis.

A structure regulation index (SRI) was introduced to quantify multiscale pore–throat evolution:

(6) $$\mathrm{SRI}=\alpha \frac{r_{mode,0}-r_{mode}}{r_{mode,0}}+\beta \frac{{\mathrm{FWHM}}_{0}-\mathrm{FWHM}}{{\mathrm{FWHM}}_{0}}+\gamma \frac{S_{BET}-S_{BET,0}}{S_{BET,0}}$$

The weighting scheme in Equation (6) reflects the relative contributions of pore–throat geometric regulation and interfacial complexity to seepage behavior. The modal throat radius (*r_mode_*) directly characterizes the characteristic size of flow-controlling throats and thus exerts a first-order control on permeability. The full width at half maximum (FWHM) describes the dispersion of the throat-size distribution; a reduction in FWHM indicates distribution convergence and suppression of preferential flow heterogeneity, which is equally critical for stabilizing dominant flow pathways. Therefore, *r_mode_* and FWHM were assigned higher weights (α = 0.4 and β = 0.4) as dominant structural descriptors. In contrast, the specific surface area (*S_BET_*) primarily reflects surface roughness and interfacial complexity, which affects flow resistance indirectly through adsorption and boundary-layer effects; its influence on permeability is secondary compared with geometric throat constraints, justifying a lower weight (γ = 0.2). Sensitivity analysis further confirmed that variations in *r_mode_* and FWHM induce stronger permeability responses than comparable changes in *S_BET_*, supporting the adopted weighting scheme.

Where the subscript “0” indicates the original parameter. Weight coefficients were assigned as α = 0.4, β = 0.4 and γ = 0.2, based on the dominant roles of pore–throat refinement and distribution convergence and the secondary influence of specific surface area, further supported by sensitivity analysis.

The influence of structure regulation on seepage behavior was evaluated using the Katz–Thompson equation:

(7) $$k\approx \frac{1}{226}r_{c}^{2}\frac{\phi^{3}}{{\left(1-\phi \right)}^{2}}$$

where $r_{c}$ is the critical throat radius and ϕ is the porosity. This model links microscale pore–throat variation to macroscale permeability, enabling cross-scale evaluation of regulatory behavior. By combining the SRI with the Katz–Thompson relationship, the multiscale regulation of seepage behavior can be explicitly interpreted. Structural refinement and distribution convergence, as captured by increasing SRI values, correspond to a systematic reduction in critical throat radius *r_c_* and effective pore connectivity, which in turn governs macroscopic permeability. This cross-scale linkage enables SRI to serve as an integrated descriptor connecting pore–throat evolution to flow behavior under gel injection, curing, and degradation processes.

#### 4.5.6. Surface Energy and Interfacial Wettability

Surface free energy (SFE) reflects the interaction capability of a solid surface and consists of a dispersive component (γ^d^) and a polar component (γ^p^). Using the Owens–Wendt method, SFE is obtained by measuring contact angles of probe liquids (e.g., water and diiodomethane).

(8) $$\gamma_{L}(1+\cos\theta )=2\left(\sqrt{\gamma_{s}^{d}\gamma_{L}^{d}}+\sqrt{\gamma_{s}^{p}\gamma_{L}^{p}}\right)$$

where γ_L_ is the surface tension of the liquid, and γ_L_^d^, γ_L_^p^ are its dispersive and polar components. By combining measurements of two liquids, the solid-surface components γ_s_^d^ and γ_s_^p^ can be obtained.

## Figures and Tables

**Table 1:** Effect of SiO_2_ Content on the Network Structural Parameters of DSRC-NCG (curing time = 1.0 h; 70 °C; salinity = 30.0 g/L).

SiO_2_ (wt%)	*d*_f_ (nm)	*ρ*_node_ (μm^−2^)	*ξ* (nm)	*D*c (nm)	Ra (nm)
0.08	118.6	0.94	410	1.2	9.8
0.12	97.3	1.41	340	1.5	11.3
0.16	82.5	1.98	275	1.8	13.7
0.20	78.9	2.15	260	1.9	14.9
0.22	81.7	2.08	265	2.0	15.2
0.28	95.4	1.52	320	2.4	18.4

**Table 2:** Effect of Curing Time on the Network Structural Parameters of DSRC-NCG (SiO_2_ = 0.20 wt%; 70 °C; salinity = 30.0 g/L).

Curing Time (h)	*d*_f_ (nm)	*ρ*_node_ (μm^−2^)	*ξ* (nm)	*D*c (nm)	Ra (nm)
0.5	104.2	1.36	335	1.6	11.0
0.8	89.3	1.88	285	1.8	13.2
1.0	78.9	2.15	260	1.9	14.9
1.5	76.5	2.21	255	2.0	15.1
2.0	77.1	2.19	258	2.1	15.0
3.0	83.6	2.02	275	2.2	14.2

**Table 3:** Specific surface area, pore volume, and average pore size of DSRC-NCG with different SiO_2_ contents.

SiO_2_ (wt%)	Specific Surface Area (m^2^/g)	Pore Volume (cm^3^/g)	Average Pore Size (nm)
0.08	112.4	0.382	13.6
0.12	128.7	0.426	12.5
0.16	146.3	0.471	11.8
0.20	152.9	0.495	11.2
0.24	138.5	0.452	12.9

**Table 4:** Connectivity index and dominant-channel characteristics of DSRC-NCG with different SiO_2_ contents.

SiO_2_ (wt%)	Connectivity Index (CI)	Dominant-Channel Proportion (%)	Average Channel Length (μm)
0.08	0.46	34.2	18.6
0.12	0.52	39.7	20.3
0.16	0.61	46.5	22.8
0.20	0.67	51.9	24.1
0.24	0.55	42.3	19.7

**Table 5:** Surface chemical and interfacial characteristics of DSRC-NCG with different SiO_2_ contents.

SiO_2_ (wt%)	FTIR Peak Position (C=O, cm^−1^)	Si–O–Si Peak Intensity (a.u.)	O/C Atomic Ratio (XPS)	Contact Angle (°)
0.08	1731.4	0.84	0.36	92.5
0.12	1728.7	0.97	0.42	87.6
0.16	1726.8	1.15	0.48	81.3
0.20	1726.1	1.24	0.51	78.4
0.24	1727.5	1.10	0.47	83.2

**Table 6:** Surface free energy parameters of DSRC-NCG calculated using the Owens–Wendt model.

SiO_2_ (wt%)	θ_water_ (°)	θ_DIM_ (°)	γ^d^ (mJ/m^2^)	γ^p^ (mJ/m^2^)	γ^tot^ (mJ/m^2^)
0.08	87.5	52.3	28.7	6.4	35.1
0.12	82.4	49.8	29.1	8.9	38.0
0.16	78.9	47.6	29.5	11.8	41.3
0.20	74.6	45.2	30.2	13.6	43.8
0.24	77.8	47.1	29.4	11.2	40.6

**Table 7:** Rheological and mechanical performance parameters of DSRC-NCG with different SiO_2_ contents.

SiO_2_ (wt%)	Viscosity *η* (Pa·s, 10 s^−1^)	G′ (Pa, 1 Hz)	G″ (Pa, 1 Hz)	G′/G″	Modulus Retention (%)
0.08	12.4	1860	1250	1.49	72.5
0.12	18.7	2540	1395	1.82	81.3
0.16	27.9	3680	1520	2.42	88.6
0.20	32.5	4120	1610	2.56	91.7
0.24	29.1	3650	1680	2.17	85.2

**Table 8:** Injectivity index and pressure-response parameters of DSRC-NCG (repeated injections under identical 1.0 mD core conditions).

SiO_2_ (wt%)	Initial J (mL·h^−1^·kPa^−1^)	Steady-State J (mL·h^−1^·kPa^−1^)	Peak Pressure (kPa)	Pressure Half-Decay Time (h)
0.08	0.162	0.153	23.6	0.42
0.12	0.149	0.139	28.4	0.56
0.16	0.134	0.125	33.1	0.73
0.20	0.129	0.121	35.7	0.81
0.24	0.138	0.130	31.5	0.60

**Table 9:** In situ micro-CT sequential network parameters (same core, SiO_2_ = 0.20 wt%).

Stage	T (h)	φ_c_ (%)	*V*_dom_ (%)	rth (μm)	Z (−)	*τ* (−)	*χ* (−)	*k*_img_ (mD)	*k*_exp_ (mD)
Baseline (0 h)	0.0	11.2	7.6	0.36	2.42	1.78	−102	0.52	0.50
0.3 PV	0.3	12.1	9.4	0.31	2.71	1.63	−88	0.31	0.30
0.6 PV	0.6	12.9	10.7	0.28	2.96	1.55	−75	0.22	0.21
Hold 2.0 h	2.0	13.1	11.0	0.27	3.04	1.52	−72	0.20	0.19
Degrade 12 h	12.0	12.5	9.8	0.30	2.83	1.58	−81	0.36	0.35
Degrade 24 h	24.0	11.8	8.2	0.34	2.55	1.66	−95	0.44	0.43

Notes: *φ*_c_ = connected porosity; *V*_dom_ = volume fraction of dominant flow channels; *τ* = shortest-path length divided by Euclidean distance; *χ*: more negative values indicate fewer isolated cavities and more continuous channels; *k*_img_ = permeability estimated using LBM inversion; *k*_exp_ = permeability obtained by steady-state measurement.

**Table 10:** Effect of SiO_2_ content on network parameters (blocking stage, 2.0 h).

SiO_2_ (wt%)	*V*_dom_ (%)	*Z* (−)	*τ* (−)	rth (μm)	*χ* (−)	*k*_img_ (mD)	*k*_exp_ (mD)
0.12	8.4	2.55	1.67	0.34	−97	0.41	0.40
0.16	10.2	2.86	1.56	0.30	−82	0.29	0.28
0.18	11.5	3.02	1.51	0.28	−74	0.23	0.22
0.20	11.8	3.08	1.49	0.27	−71	0.20	0.19
0.22	11.0	2.97	1.52	0.27	−76	0.22	0.21
0.24	9.6	2.71	1.60	0.25	−89	0.28	0.27
0.28	7.9	2.38	1.74	0.24	−121	0.39	0.38

**Table 11:** Statistical pore–throat structural parameters before and after gel treatment (combined MICP–BET characterization).

Group	Threshold Pressure P_c,th_ (MPa)	Dominant Throat Radius r_mode_ (μm)	FWHM (MICP)/μm	BI	S_BET_ (m^2^/g)	Mesopore Peak r_meso,mode_ (nm)	FWHM (BJH) (nm)	SRI
Untreated	2.35	0.353	0.212	0.08	7.42	11.82	5.62	0
0.12 wt%	2.68	0.308	0.187	0.10	8.15	10.73	5.27	0.17
0.16 wt%	3.12	0.271	0.171	0.09	8.94	9.86	4.81	0.29
0.20 wt%	3.36	0.248	0.163	0.10	9.18	9.62	4.56	0.33
0.24 wt%	3.40	0.236/0.381	0.272	0.34	9.02	9.47/12.21	6.13	0.05
After degradation	2.52	0.331	0.218	0.09	7.88	11.33	5.51	0.02

**Table 12:** Composition and functional roles of the DSRC-NCG gel system.

Component Category	Representative Material	Functional Role in DSRC-NCG
Polymer backbone	Acrylamide-based copolymer	Formation of the primary gel network and mechanical framework
Supramolecular unit	β-Cyclodextrin-grafted polymer	Host sites for reversible supramolecular crosslinking
Supramolecular unit	Adamantane-grafted polymer	Guest moieties enabling dynamic host–guest interactions
Nanofiller	Amine-functionalized SiO_2_ nanoparticles	Network reinforcement and pore-scale structural regulation
Ionic environment	NaCl/CaCl_2_ aqueous solution	Simulation of high-salinity reservoir conditions
Porous media	Artificial tight sandstone cores	Core-scale flow and permeability evaluation
Pore-scale model	Glass microfluidic chips	Visualization of pore-flow interaction mechanisms

## Data Availability

The figures and tables used to support the findings of this study are included in the article.

## Appendix A

**Table A1:** Abbreviations used in this study.

Abbreviation	Full Name	Description
DSRC-NCG	Dual-Structure-Regulated Nanocomposite Gel	Nanocomposite gel system investigated in this study
SRI	Structural Regulation Index	Index describing multiscale pore-throat structural regulation
HCI	Pore–Flow Matching Index	Index quantifying pore–flow matching across permeability levels
RRF	Residual Resistance Factor	Ratio of permeability before and after gel treatment
*f*_dom_	Dominant-channel fraction	Fraction of dominant flow channels
J	Injectivity index	Indicator describing gel injectivity performance

**Table A2:** Symbols and parameters.

Symbol	Description	Unit
*d*_f_	Fiber diameter of gel network	nm
*ρ*_node_	Node density of gel network	μm^−2^
*ξ*	Mesh characteristic length	nm
Ra	Arithmetic surface roughness (AFM)	nm
*η*	Apparent viscosity (10 s^−1^)	Pa·s
G′	Storage modulus	Pa
G″	Loss modulus	Pa
*σ*	Compressive strength	MPa
*E*	Elastic modulus	MPa
*ε*_f_	Failure strain	%
*SR*	Swelling ratio	g/g
*ϕ*_c_	Connected porosity	%
*V*_dom_	Dominant-channel volume fraction	%
rth	Average throat radius	μm
*Z*	Coordination number	–
*τ*	Tortuosity	–
χ	Euler characteristic	–
*k*_img_	Image-based permeability	mD
*k*_exp_	Experimental permeability	mD
*K*	Core permeability	mD
